# Case Report: Identification of a *de novo* Missense Mutation in the *F8* Gene, p.(Phe690Leu)/c.2070C > A, Causing Hemophilia A: A Case Report

**DOI:** 10.3389/fgene.2020.589899

**Published:** 2021-03-05

**Authors:** Haiyan Bai, Xia Xue, Li Tian, Xi Tong Liu, Qian Li

**Affiliations:** Assisted Reproductive Center, Women’s & Children’s Hospital of Northwest, Xi’an, China

**Keywords:** hemophilia A, preimplantation genetic testing for monogenic disease, p.(Phe690Leu)/c.2070C > A, next-generation sequencing, chromosome copy number variation, single nucleotide polymorphism

## Abstract

Hemophilia A is an X-linked recessive bleeding disorder caused by various types of pathological defects in the factor VIII gene (*F8/*FVIII). Preimplantation genetic testing for monogenic disease (PGT-M) is a powerful tool to tackle the transmission of monogenic inherited disorders from generation to generation. In our case, a mutation in *F8* had passed through female carriers in a hemophilia A family and resulted in two male patients with hemophilia A. To identify the etiological genetic variants of *F8*, next-generation sequencing (NGS) was used for chromosome copy number variation detection, Sanger sequencing to verify mutation sites, single nucleotide polymorphism (SNP) for site amplification, and sequencing to validate the genetic linkage. Finally, a novel missense mutation, p. (Phe690Leu)/c.2070C > A, occurring in exon 13 of *F8*, was screened out as a pathogenic mutation. Following this, an *F8* normal euploid blastocyst was transferred. At the 18th week, the pregnant mother underwent amniocentesis, NGS, Sanger sequencing, and SNP typing that further confirmed that the fetus had a healthy genotype. After delivery, a neonatal blood sample was sent for FVIII concentration detection, and the result established that the FVIII protein was rescued to a nearly average level. We first identified a new type of pathogenic mutation in *F8*, which has not been previously reported, selected a genetically healthy progeny for an affected family, and provided valuable knowledge of the diagnosis and treatment of hemophilia A.

## Introduction

Hemophilia A (OMIM 306700) is an inherited X-linked recessive bleeding disorder. It is caused by the deficiency of blood coagulant activities of factor VIII (FVIII), due to abnormalities in the *F8* coding gene (Keeney et al., 2005). The father with a homozygous mutation will certainly pass the mutation to a female child who would phenotypically be a normal female hemizygous carrier, whereas none of the male children born will be affected. A mother with homozygous mutation will give birth to male children with homozygous mutation and a has a 50% risk of giving birth to a hemizygous carrier female child. Both male and female descendants could be affected by the disease if both parents are homozygous for the abnormal gene. Therefore, hemophilia A rarely occurs in female individuals, as it always appears in a heterozygote form. Conversely, the incidence is estimated to be 1:5,000–10,000 in male individuals (Hallden et al., 2012). Activity and circulating plasma levels of FVIII protein are used to evaluate the severity of hemophilia A, which can be classified as severe, moderate, and mild, corresponding to FVIII protein levels of ≤1, 2–5, and 5–30%, respectively (Srivastava et al., 2013).

The *F8* gene (MIM + 300841) is located at the distal end of the long arm of the X chromosome (Xq28), which is 186 kb in size [hg19: chrX:154064064-154250998; UCSC genome browser^1^], comprising 26 exons and 25 introns. Since the first publication of the *F8* sequence in 1984, more than 2,000 gene mutations corresponding to 5,472 individual case reports of hemophilia A have been reported. These mutations are described in the Human Gene Mutation Database (HGMD^2^) and Factor VIII Variant Database^3^. Currently, different heterogeneous genetic mutations in the *F8* gene cause hemophilia A, including inversion in intron 22 and intron 1, point mutations, small deletions/insertions/duplications, and large deletions/duplications. Point mutations caused by missense mutations usually result in 57 mild or moderate illness. Other types of variants always cause severe outcomes (Peyvandi et al., 2013; Lannoy and Hermans, 2016). Therefore, the identification of *F8* mutations can be crucial in genetic counseling and prenatal diagnosis.

In this study, with the use of next-generation sequencing (NGS), Sanger sequencing, and single nucleotide polymorphism (SNP) typing, we reported the identification and characteristics of a novel missense mutation (p.(Phe690Leu)/c.2070C > A) occurring in exon 13 of *F8* in a hemophilia A family, which caused the disease. This mutation has not been previously reported in HGMD and PubMed.

## Case Description

### Assessment of Family F8 Mutation

**FIGURE 1:**
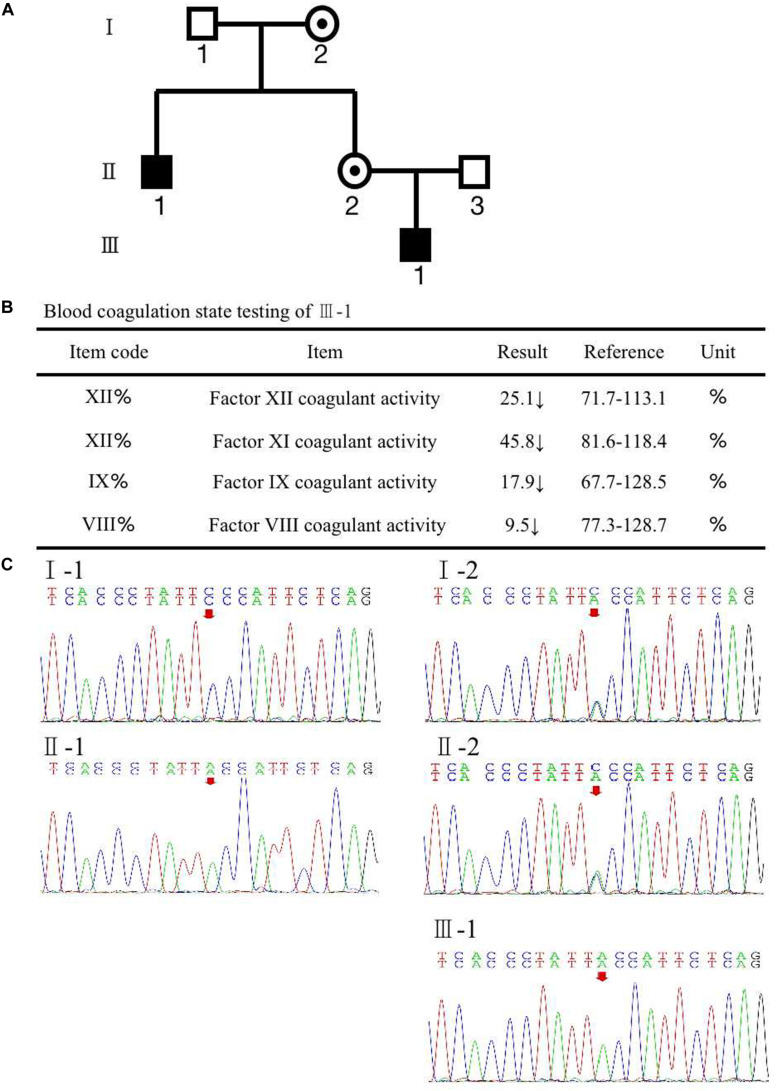
**(A)** Pedigree of the family. The white square represents healthy male individuals; the black square indicates affected male individuals; the black circle with a dot in the middle indicates carrier female individuals. **(B)** The inspection report of III-1. **(C)** Mutation screening of the principle of family members using Sanger sequencing. The red arrow indicates the mutation site of (Phe690Leu)/c.2070C > A of *F8* at an individual person.

A woman (II-2) presented with no apparent clinical phenotype of hemophilia A came to our assisted reproduction center for PGT-M counseling because her son (III-1) and her brother (II-1) were both affected by hemophilia A (Figure 1A). The activated partial thromboplastin time (APTT) in her son was 121.5 s, and FVIII coagulant activity was 9.50%, corresponding to mild hemophilia A. The hemophilia A patients suffered from recurrent hemarthrosis and continuous bleeding after an injury (Figure 1B and Supplementary Figure S1).

To recognize the potential etiological genetic variants, we screened the *F8* gene in her family. The first stage was the pedigree pretest. Genomic DNA was isolated from blood samples based on the principle of family members. Sanger sequencing was used to screen for *F8* mutations in these family members. The result is shown in Figure 1C; a variation, c.2070C > A in exon 13 of *F8*, was found in the mother (II-2). The results also revealed that the mutated nucleotide A was acquired from the II-2’s mother (I-2) and therefore passed to the brother (II-1), the mother herself (II-2), and the mother’s son (III-1). The father and husband of II-2 (I-1 and II-3) had a normal *F8* gene. II-2 and her mother were heterozygous for the mutation without disease phenotype. However, her brother and her son were homozygotes for the mutation, and both were affected by hemophilia A. The mutation results in an amino acid change of phenylalanine to leucine (p. Phe690Leu). The current knowledge of this kind of mutation suggests that it may have an impact on the configuration and conformation of FVIII protein, leading to the loss of its function. This is likely to be a pathogenic cause of hemophilia A (Gouw et al., 2012; Chen et al., 2016; Stephensen et al., 2019).

### PGT-M Procedure

**FIGURE 2:**
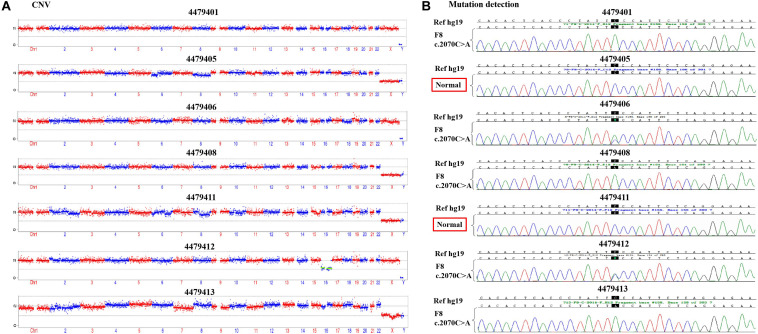
**(A)** The copy number variation (CNV) detection and **(B)** the mutation screening of the seven blastocysts.

According to the mother-II-2’s wish, we performed intracytoplasmic sperm injection with PGT-M for her assisted reproduction trial. Finally, seven blastocysts (4479401, 4479405, 4479406, 4479408, 4479411, 4479412, and 4479413) were obtained, and trophectoderm biopsy was performed and sent for copy number variation (CNV) testing by NGS, mutation detection through Sanger sequencing, and SNP site amplification and sequencing. First, all blastocysts detected without CNV at 4 M resolution level except for embryo 4479412 [45, XN, -16(× 1)] lost chromosome 16 (Figure 2A). Second, after Sanger sequencing, only two out of the seven blastocysts were detected as normal as c.2070C > C in exon 13 of *F8* (Figure 2B). Furthermore, SNP typing validated that the two euploid and non-mutation embryos detected in the first two steps inherited the normal X chromosome from their mother (Table 1). The two embryos were numbered 4479405 and 4479411 (highlighted by the red rectangular box or red color), and both were male. Embryos 4479401 and 4479406 were female, and embryo 4479408 and 4479413 were male. These four embryos inherited SNP abnormal chromosome X from the mother (II-2), consistent with the Sanger result and thus were excluded.

**TABLE 1:** Single nucleotide polymorphism (SNP) typing of the family and preimplantation genetic testing for monogenic disease (PGT-M) embryos.

**SNP Number**	I**-1**	I**-2**	II**-1**	II**-2 normal**	II**-2 carry**	**4479401**		**4479405**	**4479406**		**4479408**	**4479411**	**4479412**		**4479413**
	**Haplotype**	**Haplotype**	**Haplotype**	**Haplotype**	**Haplotype**	**Maternal haplotype**	**Maternal haplotype**	**Maternal haplotype**	**Paternal haplotype**	**Maternal haplotype**	**Maternal haplotype**	**Maternal haplotype**	**Paternal haplotype**	**Maternal haplotype**	**Maternal haplotype**
	
YK-F8-SNP01	T	T/C	C	T	C	C	C	T	C	C	C	T	C	C	C
YK-F8-SNP02	C	C/A	A	C	A	A	A	C	A	A	A	C	A	A	A
YK-F8-SNP07	C	A/A	A	C	A	A	A	C	A	A	A	C	A	A	A
YK-F8-SNP13	C	T/T	C	C	T	T	T	C	C	T	T	C	C	T	T
YK-F8-SNP14	T	C/C	T	T	C	C	C	T	T	C	C	T	T	C	C
YK-F8-SNP15	C	G/C	C	C	G	G	G	C	C	G	G	C	C	G	G
YK-F8-SNP16	T	C/C	T	T	C	C	C	T	T	C	C	T	T	C	C
YK-F8-SNP17	T	G/G	G	T	G	G	G	T	G	G	G	T	G	G	G
YK-F8-SNP18	T	C/C	C	T	C	C	C	T	C	C	C	T	C	C	C
YK-F8-SNP20	T	A/A	T	T	A	A	A	T	T	A	A	T	T	A	A
YK-F8-SNP22	C	C/A	C	C	A	A	A	C	C	A	A	C	C	A	A
YK-F8-SNP23	C	T/T	C	C	T	T	T	C	C	T	T	C	C	T	T
YK-F8-SNP25	C	G/G	C	C	G	G	G	C	C	G	G	C	C	G	G
YK-F8-SNP27	A	G/A	A	A	G	G	G	A	A	G	G	A	A	G	G
YK-F8-SNP32	T	C/T	C	T	C	C	C	T	C	C	C	T	C	C	C
YK-F8-SNP33	T	C/T	C	T	C	C	C	T	C	C	C	T	C	C	C
YK-F8-SNP34	G	G/T	T	G	T	T	T	G	T	T	T	G	T	T	T
YK-F8-SNP35	C	C/T	T	C	T	T	T	C	T	T	T	C	T	T	T
YK-F8-SNP37	G	G/A	A	G	A	A	A	G	A	A	A	G	A	A	A
YK-F8-SNP38	A	A/C	C	A	C	C	C	A	C	C	C	A	C	C	C
YK-F8-SNP39	C	T/C	T	C	T	T	T	C	T	T	T	C	T	T	T
YK-F8-SNP40	T	C/C	T	T	C	C	C	T	T	C	C	T	T	C	C
YK-F8-SNP42	G	A/A	A	G	A	A	A	G	A	A	A	G	A	A	A
YK-F8-SNP44	A	G/G	A	A	G	G	G	A	A	G	G	A	A	G	G
YK-F8-SNP45	C	G/C	C	C	G	G	G	C	C	G	G	C	C	G	G
YK-F8-SNP46	T	C/T	T	T	C	C	C	T	T	C	C	T	T	C	C
YK-F8-SNP47	T	T/A	T	T	A	A	A	T	T	A	A	T	T	A	A
YK-F8-SNP48	T	T/C	T	T	C	C	C	T	T	C	C	T	T	C	C
YK-F8-SNP49	C	G/G	C	C	G	G	G	C	C	G	G	C	C	G	G
YK-F8-SNP50	G	A/A	G	G	A	A	A	G	G	A	A	G	G	A	A
YK-F8-SNP51	G	A/A	G	G	A	A	A	G	G	A	A	G	G	A	A
YK-F8-SNP58	A	A/G	G	A	G	G	G	A	G	G	G	A	G	G	G
YK-F8-SNP61	A	A/G	G	A	G	G	G	A	G	G	G	A	G	G	G
YK-F8-SNP62	C	C/T	T	C	T	T	T	C	T	T	T	C	T	T	T
YK-F8-SNP63	A	A/G	G	A	G	G	G	A	G	G	G	A	G	G	G
YK-F8-SNP65	C	C/T	T	C	T	T	T	C	T	T	T	C	T	T	T
YK-F8-SNP66	A	A/G	G	A	G	G	G	A	G	G	G	A	G	G	G
YK-F8-SNP67	A	G/A	G	A	G	G	G	A	G	G	G	A	G	G	G
YK-F8-SNP68	C	C/T	T	C	T	T	T	C	T	T	T	C	T	T	T
YK-F8-SNP72	A	A/G	G	A	G	G	G	A	G	G	G	A	G	G	G
YK-F8-SNP73	G	A/G	A	G	A	A	A	G	A	A	A	G	A	A	A
YK-F8-SNP75	C	G/C	G	C	G	G	G	C	G	G	G	C	G	G	G
YK-F8-SNP79	G	C/C	G	G	C	C	C	G	G	C	C	G	G	C	C
YK-F8-SNP80	T	C/C	T	T	C	C	C	T	T	C	C	T	T	C	C
YK-F8-SNP81	T	C/T	T	T	C	C	C	T	T	C	C	T	T	C	C

### Amniocentesis Testing and Detection of Newborn FVIII Activity

**FIGURE 3:**
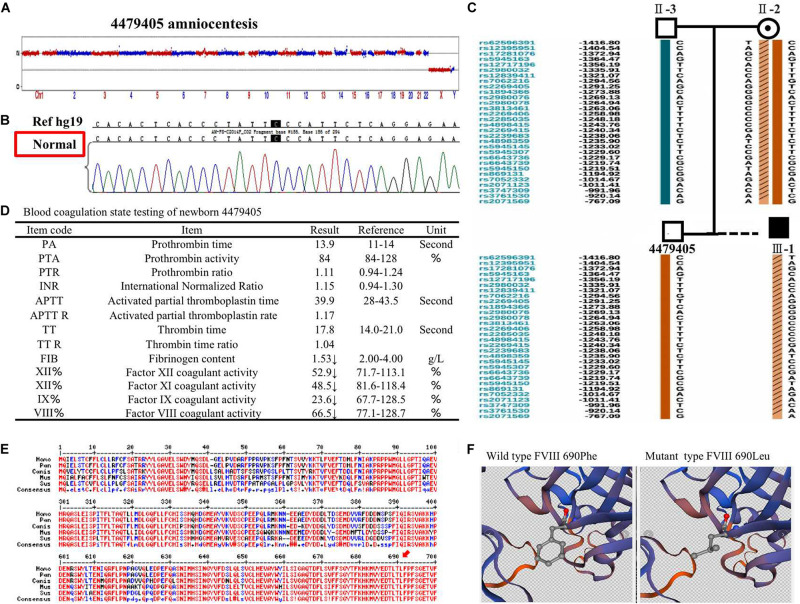
**(A)** The copy number variation (CNV), **(B)** mutation, and **(C)** single nucleotide polymorphism (SNP) detection of amniotic fluid at the 18th pregnancy week after transfer blastocyst 4479405. **(D)** The inspection report of newborn 4479405. **(E)** The consensus of 690 Phe of *F8* among different specials. **(F)** The 3D structure of wild-type FVIII and mutant FVIII. The red arrow represents the conservation of the site Phe690 of *F8* among different species.

The 4479405 embryo was selected for blastocyst transfer. At the 18th week, amniocentesis was applied to the mother-II-2 to further confirm the genetic health of the fetus. The result was the same as that in the blastocyst examination. There was no significant abnormal CNV detection at the 4 M resolution level as well as no genetic mutation (Figures 3A,B). Regarding the SNP typing, as illustrated in Figure 3C, the fetus 4479405 inherited the SNP normal X chromosome from the mother, while the previous III-1, who was affected by hemophilia A, inherited the X chromosome with the pathogenic SNP.

After delivery, the FVIII concentration of newborn 4479405 was analyzed after 1 month. The result is shown in Figure 4A. The FVIII activity was rescued to 66.5% compared with the 9.50% of the first child (III-1) with hemophilia A of the mother-II-2. According to the doctor’s opinion, FVIII activity became almost normal (Figure 3D and Supplementary Figure S2). These results showed that the mutation site p. (Phe690Leu)/c.2070C > A was exactly the etiological site responsible for hemophilia A. The protein conservation of Phe690 of F8 showed high consensus among *Homo sapiens*, *Pan troglodytes*, *Canis lupus familiaris*, *Mus musculus*, and *Sus scrofa*; high consensus was labeled as red color (Figure 3E, arrow). The possible damaging effect of the mutation to FVIII protein 3D structure and software-predicted damaging parameters are illustrated in Figure 3F and listed in Supplementary Figure S3. Compared with wild-type FVIII, the mutant 690 Leu FVIII lacks the benzene ring of the original phenylalanine; the bioinformatic parameters all indicate that such change has harmful effect on FVIII protein.

## Discussion

Due to the X-linked recessive mode of inheritance, hemophilia A usually affects male individuals, while the female family members commonly present as deficient gene carriers who may pass the mutation gene to their progeny (Lacroix-Desmazes et al., 2020; Ling and Tuddenham, 2020).

Based on the present report, we detected a novel missense mutation occurring in exon 13 of the *F8* gene, p. (Phe690Leu)/c.2070C > A. In the inspected family, this novel mutation had been passed on by the phenotypically normal heterozygote female family members (I-2 and II-2). Male descendants were all affected by inheriting their mother’s deficient gene (II-1 and III-1). In the PGT-M treatment cycle of mother-II-2, two out of the seven embryos were confirmed as euploid as well as possessing a non-mutated *F8* gene through CNV testing, mutation detection, and SNP typing. The genetic selection of embryo transfer led to an *F8* normal fetus as seen at the 18th week. Finally, the FVIII concentration was rescued to nearly normal levels after the baby was born. Through PGT-M diagnosis and embryo selection, we found a novel *F8* mutation and ensured that a normal *F8* gene and a healthy CNV were present. The outcome provided us with the confidence that the mutation we found was pathogenic, which is relevant to hemophilia A knowledge.

One of the major genetic challenges is to distinguish causal mutations from polymorphisms. The mutation c.2070C > A in exon 13 was not registered in the SNP database (dbSNP) and has not been reported in HGMD and PubMed. Thus, the mutation was recognized as a novel *F8* mutation. This type of mutation may be induced during the normal process of DNA replication, termed a replication error, which occurs in a remarkably low frequency, or may be caused by cell metabolism through depurination/depyrimidination and deamination, called a spontaneous lesion. It was estimated that it occurs in 10,000–1,000,000 nucleotides per cell per day (de Ligt et al., 2013; Lannoy and Hermans, 2016).

The effect of p. (Phe690Leu)/c.2070C > A mutation on FVIII protein was confirmed by bioinformatics analysis (Figures 3E,F and Supplementary Figure S3). It is generally assumed that the more conservative the locus is, the more likely it is to cause harmful results when it is mutant. The Phe690 site of FVIII protein was highly conserved among different specials, which means that it has a great possibility to affect protein function when changed. In a previous study, the mutation of Phe to Leu in allene oxide synthase (AOS) changes its catalysis activity to epoxyalcohol synthase (EAS), which is another fatty acid hydroperoxide metabolizing enzymes of the CYP74 family (Toporkova et al., 2020). In another study, the mutation of Phe to Leu in the 3C-like protease (3CLpro) of severe acute respiratory syndrome coronavirus (SARS-CoV) leads to conformational change and autoinhibition of the enzyme (Muramatsu et al., 2016). In our report, all bioinformatic software parameters indicate that the mutation we found has detrimental effect on protein function.

From the blood coagulation state testing of mother II-2’s first children affected with hemophilia A and 4479405 (Figures 1B, 3D), we can see that not only factor VIII decreased but also factor IX apparently declined, which is responsible for hemophilia B. FIX is a serine protease that plays a vital role in the coagulation cascade. Its cofactor, FVIII, is critical for FIX enzymatic activity. They cooperate in coagulation. Therefore, mutations in either protein may lead to lack of coagulation activity in the other, resulting in frequent spontaneous bleeds in patients (Nienhuis et al., 2017).

The strength of this report is to find out a confirmed *de novo* F8 gene mutation that is related to hemophilia A. The limitation is that the mutation we found is only responsible for mild hemophilia A; this depends on the patient we met with. However, whether the mutation also contributes to severe hemophilia A, that we do not know.

## Conclusion

In conclusion, in this case, the F8 gene mutation-causing hemophilia A has affected two male patients in a family, which was found out through CNV detection by NGS, mutation sites verification by Sanger sequencing, and genetic linkage analysis by SNP. Finally, a novel missense mutation, p. (Phe690Leu)/c.2070C > A, occurring in exon 13 of F8, was screened out as a pathogenic mutation. Following blastocyst transfer of the non-mutation euploid embryo, at the 18th week, the pregnant mother underwent amniocentesis; genetic testing further confirmed that the fetus had a healthy genotype. After delivery, blood testing proved that the FVIII protein has rescued to a nearly average level. This case reveals a new F8 mutation, which provides valuable knowledge for genetic counseling and treatment decisions.

## Data Availability Statement

All data and material generated or analyzed during this study are included in this published article.

## Ethics Statement

The studies involving human participants were reviewed and approved by Northwest Women’s and Children’s Hospital. The patients/participants provided their written informed consent to participate in this study. Written informed consent was obtained from the authors and participated patients for the publication of any potentially identifiable images or data included in this article.

## Author Contributions

HB was in charge of genetic consultation, diagnostic evaluation, and clinical management of the couple and wrote parts of the manuscript. XX participated in the laboratory procedures. LT and XL provided clinical assistance. QL was responsible for the literature review, writing, and coordinating the submission of the manuscript. All authors contributed to the article and approved the submitted version.

## Conflict of Interest

The authors declare that the research was conducted in the absence of any commercial or financial relationships that could be construed as a potential conflict of interest.

## Footnotes

1 http://genome.ucsc.edu/

2 http://www.hgmd.cf.ac.uk/ac/index.php

3 http://www.factorviii-db.org/
