# Supplementary material for: Case Report: Identification of a de novo Missense Mutation in the F8 Gene, p.(Phe690Leu)/c.2070C > A, Causing Hemophilia A: A Case Report
Source: Front Genet. 2021 Mar 5;11:589899. doi: 10.3389/fgene.2020.589899 (PMC7973284; doi:10.3389/fgene.2020.589899)
Supplement: Supplementary file 1 [file Table_1.DOCX]

**Figure S1**

**
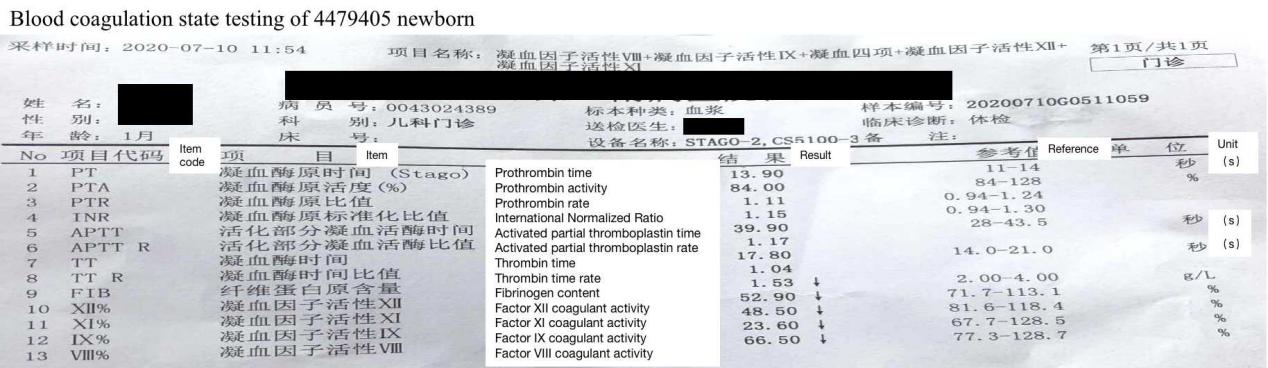
**

**Figure S2**

**
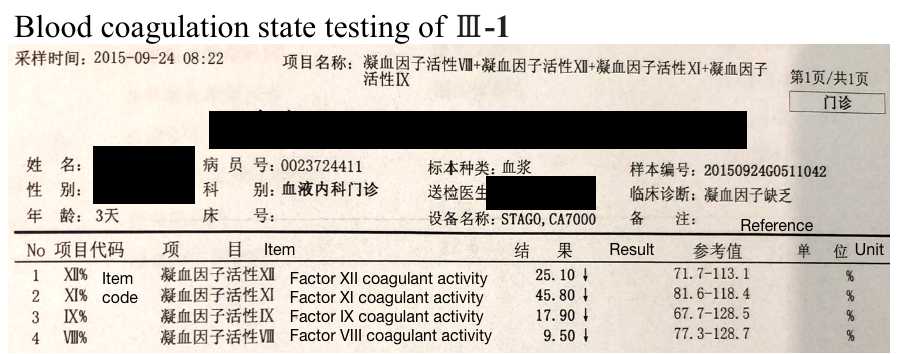
**

**Figure S3**

| **Overview of the F8** mutation |  |  |
| --- | --- | --- |
|  |  | Ref range |
| cDNA change | c.2070C>A |  |
| Protein change | p.(Phe690Leu) |  |
| Mutation type | Missense |  |
| SIFT | 0.012 | <0.05 damaging |
| PROVEAN | -4.99 | ≤-2.5 damaging |
| Polyphen-2-HumVar | 0.994 | 0-1 |
| MutationTaster | 0.749 | 0-1 |
| CADD_phred | 26.2 | >15 |
| SIFT, PROVEA and Polyphen-2-HumVar represent the effect of the variation on the protein sequence; | | |
| MutationTaster indicate the conservation of the gene locus, the closer to 1, the more conserved the gene locus is; | | |
| CADD_phred score the harmfulness of SNV and InDel. | | |
